# Toxic inhalation of sodium metabisulphite by-products from a shipping container

**DOI:** 10.1093/occmed/kqad009

**Published:** 2023-02-17

**Authors:** C Sack, C K Reeb-Whitaker, D Todorov, P S Darby

**Affiliations:** Department of Medicine and Department of Environmental and Occupational Health Sciences, The University of Washington, Seattle, WA 98195, USA; Washington State Department of Labor and Industries, Safety and Health Assessment and Research for Prevention (SHARP) Program, Olympia, WA 98501, USA; Washington State Department of Labor and Industries, Safety and Health Assessment and Research for Prevention (SHARP) Program, Olympia, WA 98501, USA; Virginia Mason Franciscan Occupational Medicine Associates, Tacoma, WA 98003, USA; Department of Environmental and Occupational Health Sciences, University of Washington, Seattle, WA 98195, USA

## Abstract

Logistics workers who handle cargo containers are at risk of toxic inhalation injuries, although prevalence and severities of these injuries are not well characterized. We report on a previously healthy 37-year-old supervisor who was acutely exposed to sodium metabisulphite and its thermal degradation by-products during a routine inspection of a shipping container. The employee developed chemical pneumonitis with acute non-cardiogenic pulmonary oedema and subsequent severe reactive airway dysfunction syndrome.

## Teaser

Exposure to the by-products of sodium metabisulphite, a dry chemical shipped as bulk cargo, resulted in a life-changing toxic inhalation injury for a port logistics worker. This case adds to the growing body of reports documenting the hazardous exposures that may occur when workers open shipping containers. Procedures to open shipping containers safely are needed to protect worker health.

**Key learning points**

**What is already known about this subject:**

- Workers who handle shipping containers are at risk of exposure to respiratory hazards, including fumigants, preservatives, packing material and cargo by-products.

**What this study adds:**

- To the authors’ knowledge, this is the first reported case of inhalational injury due to sodium metabisulphite by-products from a cargo container.
- While exposure duration was brief, the worker developed severe chemical pneumonitis and subsequent reactive airway dysfunction syndrome.
- Safety awareness and prevention in the shipping industry is highly relevant today, given the ongoing pressure and constriction faced by workers throughout the global supply chain.

**What impact this may have on practice or policy:**

- More research is needed to characterize the prevalence of toxic inhalation injury in logistics workers.
- Employers should educate and train workers to anticipate and recognize potential chemical hazards and to encourage the reporting of incidents.
- Practicing the safety and health hierarchy of controls is needed to prevent serious toxic inhalation injuries in the shipping industry.

## Background

An estimated 80–90% of the world’s goods are transported by sea, with the majority packed in shipping containers. Workers who handle these containers may be unaware of potential exposures and their risk of toxic inhalation to multiple respiratory hazards, including fumigants, pesticides and cargo decomposition products. Workers may be unaware of these potential exposures and their risk of toxic inhalation. In this case study, we review the respiratory sequelae from a unique exposure to sodium metabisulphite (SMBS) by-products. SMBS, also known as sodium pyrosulfate or disodium metabisulphite, is an inorganic compound with myriad uses including as a disinfectant, antioxidant and preservative agent. His case will enhance the recognition of respiratory hazards in the shipping industry.

## Case presentation

A 37-year-old non-smoking male supervisor at a maritime port entered a shipping container (6.1 m × 2.4 m × 2.6 m) for inspection in August 2018. At baseline, the employee was fit with a body mass index of 30 and had no significant past medical history. Specifically, he had no respiratory complaints and denied any personal or family history of environmental allergies, asthma or other lung diseases.

The full shipping container had been received through the Port of Seattle, WA, USA, approximately 2 days before being delivered empty to a transportation logistics warehouse where the incident occurred. The inspection was part of the employee’s routine duties to prepare empty containers for reuse. There was no company requirement or recommendation to wear personal protective equipment when undertaking this task. On entering the empty container, the employee saw white solid particles on the floor, smelled a pungent ‘sewer’ odour and experienced acute onset of eye irritation, cough and shortness of breath. The bill of lading indicated the cargo had been 100 bags (1000 kg each) of sodium metabisulphite 97% (SMBS, Na_2_S_2_O_5_ [CAS 7681-57-4]). The employee was therefore exposed to an unknown amount of spilt residual SMBS and likely SMBS decomposition products, including sulphur dioxide (SO_2_) and other sulphite by-products. Despite leaving the area immediately, the employee’s symptoms persisted, and he went to the emergency department. He was initially treated with nebulizers and discharged home but re-presented several hours later with severe shortness of breath and tachypnoea. He was subsequently hospitalized for 3 days, requiring non-invasive positive-pressure ventilation and regular bronchodilators for respiratory distress. Computed tomography (CT) of the chest demonstrated atelectasis, septal thickening and patchy ground glass opacities. Laboratory evaluations, including complete blood count, chemistry panel and B-type natriuretic peptide, were normal.

Following this hospitalization, the employee continued to complain of dyspnoea with minimal exertion that interfered with his daily activities. He also complained of frequent chest tightness, wheezing, productive cough with whitish sputum, hoarse voice and throat constriction. He was treated with an empiric course of high-dose prednisone 0.5 mg/kg without significant objective or subjective improvement. Pulmonary function tests obtained 2 months after the injury demonstrated severe restriction with a forced vital capacity (FVC) of 2.03 L (43% predicted), forced expiratory volume in 1 s (FEV_1_) of 1.8 L (48% predicted), normal FEV_1_/ FVC ratio of 0.83, total lung capacity (TLC) of 3.61 L (57% predicted) and a moderate reduction in diffusion capacity of 19.3 mL/min/mmHg (50% predicted). The residual volume/TLC ratio was elevated to 41 (158% predicted), indicative of air trapping and there was a positive bronchodilator response with 260-mL (16%) improvement in FEV_1_ after administration of albuterol. Follow-up high-resolution CT chest showed some minimal basilar scarring, but no interstitial lung disease, mosaicism or other evidence of air trapping. Laryngoscopy was normal without evidence of vocal cord dysfunction.

**Figure 1.**
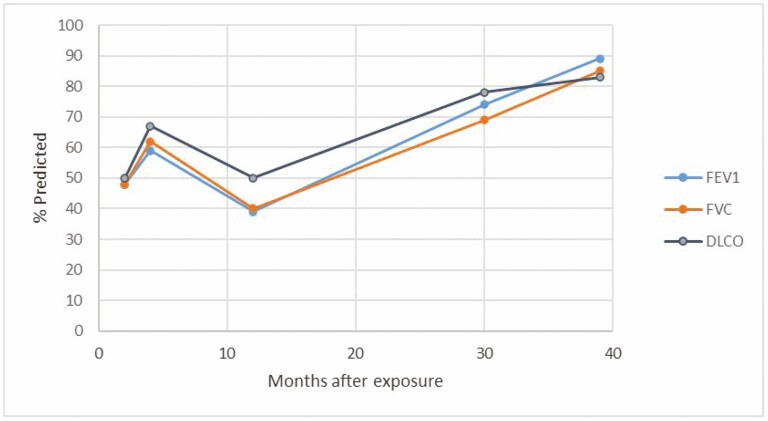
Lung function (% predicted FEV_1_, FVC, DLCO) trajectory following initial inhalational exposure. FEV_1_: forced expiratory volume in 1 s; FVC: forced vital capacity; DLCO: diffusing capacity of the lungs for carbon monoxide.

He was diagnosed with severe reactive airway dysfunction syndrome (RADS) and treated symptomatically with inhaled corticosteroids and bronchodilators. Over the course of the next three and a half years, his lung functions gradually improved to FEV_1_ of 3.43 L (89% predicted) (see Figure 1), although he continued to complain of significant symptoms and has been unable to return to work. As of November 2021, workers’ compensation claim cost was approximately US$350 200 with 3.2 time-loss years and a total permanent disability award.

This research is approved by the Washington State institutional review board; publication consent was obtained from the employee.

## Discussion

This case of toxic inhalation due to SMBS highlights the inhalation hazards associated with shipping containers. While the exposure duration was brief, the patient developed chemical pneumonitis with acute non-cardiogenic pulmonary oedema and subsequent severe RADS, functional impairment and disability.

SMBS has many chemical uses and is widely used during shipping as a preservative for fresh fruit, seafood and beverages. Inhalation of SMBS can cause acute bronchoconstriction and there are case reports of occupational asthma due to single or repetitive exposure to SMBS in the marine industry [1,2]. Additionally, there have been four fisherman deaths ascribed to the use of SMBS aboard shrimp trawlers [3]. To our knowledge, this is the first reported case of severe airway disease due to SMBS exposure from a shipping container.

Inhalation of SMBS is thought to induce injury through an irritant mechanism caused by the release of SO_2_. SMBS readily decomposes to sulphur oxides, including SO_2_, in the presence of heat, water or acid. A highly oxidative and water-soluble gas, SO_2_ is absorbed in the upper airways where it causes irritation to the conjunctiva, mucosal injury and acute bronchoconstriction [4]. Due to this immediate reaction and a low odour threshold ranging from 0.67 to 4.75 ppm for SO_2_ [5], exposed individuals may leave the area before significant damage to the respiratory tract occurs. At high concentrations, SO_2_ may cause severe airway injury with long-term pulmonary sequelae or even asphyxiation and death [6]. The National Institute for Occupational Safety and Health has established 100 ppm of SO_2_ as Immediately Dangerous to Life and Health [7].

As with most accidental exposures, the concentration of SMBS and degradation by-products, in this case, were unmeasured. The exposure was sufficient to cause chemical pneumonitis and subsequent RADS, a non-immunologic asthma-like syndrome resulting from a single, high-level exposure to an irritant gas, smoke, fume or vapour [8]. While RADS typically presents with an obstructive rather than restrictive ventilatory defect, the injured worker’s prominent asthma-like symptoms, significant air trapping and positive bronchodilator response on pulmonary function tests were consistent with the diagnosis [8]. Laryngoscopy showed no evidence of significant vocal cord damage, and a high-resolution CT scan had no mosaicism which would be the expected findings in bronchiolitis obliterans, caused by scarring of more distal small airways.

The natural history of RADS can be variable with the disease persisting for months, or years or causing permanent impairment. A case series of five miners with accidental SO_2_ exposure due to a pyrite explosion demonstrated altered bronchial response on follow-up testing 13 years after the initial injury [9]. While some of the miners demonstrated a slight improvement in spirometry in the first several weeks, none of them reached pre-accident levels [9]. While the injured worker, in this case, has demonstrated progressive improvement in his lung function, he continues to have severe respiratory symptoms and functional impairment. He remains intolerant to even minute concentrations of airborne irritants such as smoke, gasoline or household cleaners.

Toxic inhalation associated with shipping containers is most attributed to pest fumigants and volatile organic compounds; the authors found no previous reports of inhalation due to SMBS in cargo workers [10,11]. Containers may be fumigated while empty or full or may have a solid pesticide placed within them to protect the cargo during transport. The cargo itself may off-gas hazardous substances, such as carbon monoxide from wood pellets [12]; epoxies or isocyanates from polyurethane foams [13] or freshly painted surfaces (clinical experience of P.D.); and formaldehyde from processed wood [14]. The first worker to open a container for unloading may be at substantial risk of a toxic inhalation injury [15].

## Outcome and follow-up

To determine whether additional toxic inhalations were occurring in our state ports, we queried workers’ compensation claims filed with the Washington State Department of Labor and Industries. We identified seven cases of toxic inhalation related to shipping containers at warehouse and receiving yards for the period 1995–2021. The incidents involved fumigants including methyl bromide (*n* = 3), unnamed pesticides (*n* = 2), hydrogen chloride fume (*n* = 1) and unknown chemical fumes (*n* = 1). Each exposed worker sought medical care for their toxic inhalation; none involved time loss from work or disability. One limitation of workers’ compensation data is under-reporting; these seven cases likely underestimate the true burden of these injuries.

This case documents exposure to SMBS by-products and expands the knowledge of chemical exposures in the shipping industry. The severe health outcome underscores the inhalation risk that logistics workers face.
